# Advancing stem cell therapy from bench to bedside: lessons from drug therapies

**DOI:** 10.1186/s12967-014-0243-9

**Published:** 2014-09-04

**Authors:** Thekkeparambil Chandrabose Srijaya, Thamil Selvee Ramasamy, Noor Hayaty Abu Kasim

**Affiliations:** Department of Restorative Dentistry, Faculty of Dentistry, University of Malaya, Kuala Lumpur, Malaysia; Department of Molecular Medicine, Faculty of Medicine, University of Malaya, Kuala Lumpur, Malaysia

**Keywords:** Cell therapy, Drug therapy, Induced pluripotent stem cells, Mesenchymal stem cells, “Off-the-shelf” stem cell product, Stem cell homing, Stem cell migration

## Abstract

The inadequacy of existing therapeutic tools together with the paucity of organ donors have always led medical researchers to innovate the current treatment methods or to discover new ways to cure disease. Emergence of cell-based therapies has provided a new framework through which it has given the human world a new hope. Though relatively a new concept, the pace of advancement clearly reveals the significant role that stem cells will ultimately play in the near future. However, there are numerous uncertainties that are prevailing against the present setting of clinical trials related to stem cells: like the best route of cell administration, appropriate dosage, duration and several other applications. A better knowledge of these factors can substantially improve the effectiveness of disease cure or organ repair using this latest therapeutic tool. From a certain perspective, it could be argued that by considering certain proven clinical concepts and experience from synthetic drug system, we could improve the overall efficacy of cell-based therapies. In the past, studies on synthetic drug therapies and their clinical trials have shown that all the aforementioned factors have critical ascendancy over its therapeutic outcomes. Therefore, based on the knowledge gained from synthetic drug delivery systems, we hypothesize that by employing many of the clinical approaches from synthetic drug therapies to this new regenerative therapeutic tool, the efficacy of stem cell-based therapies can also be improved.

## Introduction

Stem cell technology has generated a great deal of interest in this new era of medical research due to its potential application in regenerative medicine. The two main types of stem cells basically categorized are, embryonic and non-embryonic cells. Embryonic stem cells (ESCs) are derived from the inner cell mass of the blastocyst whereas, non-embryonic stem cells, mostly adult stem cells (mesenchymal stem cells (MSCs); multipotent stem cells (MPCs); progenitor cells (PC) and somatic stem cells (SSC) are specialized cells types found in various tissues within the body [1]. Recently, another type of non-embryonic stem cells, known as induced pluripotent stem cell (iPSCs) was generated through enforced expression of defined transcription factors, which reset the fate of somatic cells to an embryonic stem-cell-like state [1]. However, ethical controversy and teratoma formation of ESCs and iPSCs hamper their clinical application.

It is generally hypothesized that cell therapy using adult stem cells hold greatest prospect of changing the face of human diseases and alleviating suffering in the near future. Consequently, the therapeutic potential of adult stem cells is the main focus of scientific research; however, in parallel, the ESCs and iPSCs have also been proposed as promising candidates for future therapies due to their pluripotency and personalized therapeutic possibilities [2]. In fact, a number of useful lessons could be learned from these two parallel cell therapy paths to visualize the hurdles to overcome as we move forward these therapies.

The use of stem cell therapy for many human clinical trials is a relatively new concept in the biomedical field [2]. Since the first successful attempts on bone marrow transplant for treating leukemia [3], a number of similar trials were initiated for treating several other diseases [4–8]. After a few decades from its first demonstration of successful clinical trials, stem cells treatments now hold a promising impact to treat various degenerative and genetic diseases including certain type of cancers [9,10], neurological diseases [11], autoimmune diseases [12] restoration of sight [13], wound healing [14], cardiac diseases [15], liver diseases [16], metabolic disorders [17], spinal cord injury [18] and bone disorders [19,20]. Thus, stem cell therapy has received stunning applause from the majority of the world population, in a hope that is expected to cater the need for diseases treatment and personalized medicine.

In the near future, it is anticipated that stem cell therapy would surpass the conventional synthetic drug therapy, which is the only treatment modality currently available for many illness. A question still remains however: can stem cells themselves can be considered as a biological drug? If we consider them as a drug, in fact, both synthetic drugs and stem cells have similar purpose in their role to cure diseases. Indeed, both therapies aim to achieve the same goal, but they are unparallel in their administration and clinical handlings due to their biochemical properties and mechanism of action in delivering their curative effects. However, it provides better opportunities to yield a more fruitful outcome if certain translational clinical concepts are merged from both therapies to formulate effective treatment strategies. Thereby, we hypothesize that the approach in curing disease through biological means can be more beneficial if we could adopt certain concepts of synthetic drug therapies, which in turn would become decisive factors. In fact, the current stem cell-based therapy in clinical trials has not yet attempted many of the concepts practiced in synthetic drug delivering systems. Nonetheless, it is anticipated that by adopting some effective strategies or procedures from synthetic drug therapy into stem cell-based therapies, an increased efficacy of the treatment could be expected.

### Current challenges in developing effective stem cell therapy

At present, the therapeutic use of stem cells poses some challenges because the underlying mechanism of action of the transplanted cells are largely unknown. Indeed, a better understanding of the properties and behaviour of these stem cells and their mechanism of action may open up an avenue for development of targeted therapies for various diseases. In fact, some critical factors have to be thoroughly considered before aligning and integrating the stem cells with the host tissues for long term beneficial outcomes.

### Heterogeneity and inherent differential potency of stem cells

Stem cells for therapeutic applications can be of autologous or allogeneic sources. Within our bodies they normally reside in complex environments and constitute heterogeneous populations [21]. Neglecting cell heterogeneity is one of the major causes of error in cell therapy. Naturally, stem cells are programmed to divide continuously and remain undifferentiated if the environment permits. Thus, the cells require proper signals or cues from neighbouring cells and the microenvironment in which the cells reside. Variations in cell to cell or cell-environmental signalling responses can alter the functional pathways of these residing cells [22]. Understanding of heterogeneity and cell potency, therefore, will aid in strategic clinical trials [23]. Hence, to interpret the inherent propensity of stem cells is a prerequisite to choose the right cell for the right application and therapy.

### Heterogeneity of disease progression

Another critical area that requires intense attention and is essential to the ultimate success of cell therapies is to explore the disease progression rate for developing tailor-made therapy with long-lasting results. This requires detailed information about the disease especially degenerative disorders to offer more personalized treatment [24]. With the advent of breakthrough in regenerative medicine such as induced pluripotent stem cells (iPSCs), there is an immense opportunity to revolutionize the way human diseases are studied, especially the genetic disorders. Creating iPSCs from patients with rare and common diseases is very useful for disease modelling in providing a platform to study the disease progression and drug development to correct the disorder [24]. This approach could be very advantageous in demonstrating the underlying molecular mechanisms, as most illness are no longer considered as single disease entities but subdivided into many factors or subtypes [25]. Given the heterogeneity and complexity of the disease, the initial implication of stem cell treatment could be tailored to an illness and later to an individual context [25]. This could move therapeutic and treatment decisions in a more systematic and target oriented approach. Such attempts can be more or less, a trial-and-error process at the beginning of the treatment but could spike the clinical efficacy of this modality.

### Homing and targeted stem cell delivery

Stem cells offer exquisite cell therapy due to their environment sensing cytokine receptors enabling these cells to migrate towards gradients of chemokine secreted by damaged tissues or tumors [26–28]. The ability of stem cells to either passively home into tissue organs or actively home into diseased sites supports the rationale for the targeted delivery [26]. Along with the native stem cell homing properties, induced-homing has been exploited for more targeted therapeutic vehicle. Much of such targeting translation have been approached by altering the cells in various ways; like substituting cell membranes with appropriate receptors [26,28], exercise lipid based particles for delivering genetically modified cells [27,29,30], use of viral vectors for gene delivery [31–33] and use of antibody or peptide conjugated particles [27,34] based on therapeutic requirement to deliver the cells of interest to the target sites. Despite the various homing mechanisms, only a very small fraction of the implanted cells migrate to desired sites, with rates of engraftment depending on method of administration. Accordingly, cell targeting efforts can be enhanced by ideal physical route, utilization of physiological forces for improving cell concentration, preconditioning of cells and introduction of transgenes which can effectively activate the cell homing process [27,32,35–37]. However, additional efforts are still needed for better indication of the stem cell behaviour and the target tissue microenvironments for using such strategies singly or in combinations to deliver the best possible therapeutics in clinical practice.

### Complexity of mechanism of action of cell therapy

The key challenge in considering novel therapies is to analyze the complex biochemical and physiological events that occur during therapeutic implications, by using animal models before translating such therapies to humans [33]. Cell therapies have often been adopted into clinical practice based on their safe and reproducible beneficial outcomes [33]. In terms of clinical practice, the basic therapeutic window is the prevention or reversal of a disease progression. In cell-based therapy both this approaches involves overlapping and differing mechanisms, which should be considered for tailor-made therapy, for disease specific clinical needs [38]. Figuring out the various possible bio-therapeutics such as anti-apoptotic, immunomodulation, anti-scarring, angiogenesis, paracrine signalling, chemo attractions and release of supporting growth and differentiation factors for local stem and progenitor cells is a daunting task to define the biochemical pathways and complex inter-molecular machinery involved in cell therapy [39,40]. Embracing the complexity of the cell, requires further research focused on the underlying behaviour and functions as one of the compelling requirements of cell therapy platform to improve the predictive power of stem cells [40,41].

### Hypothesis

**Figure 1:**
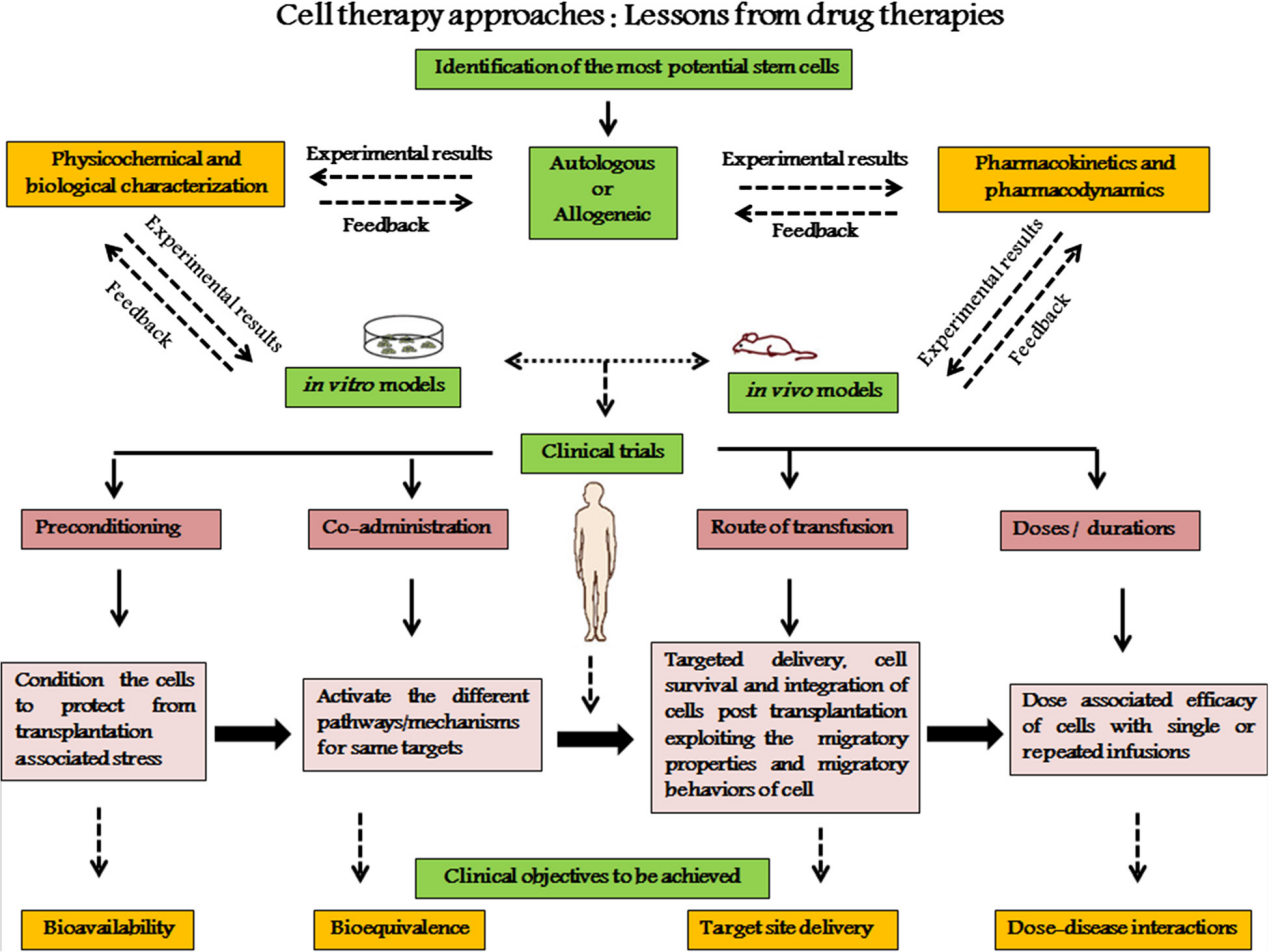
**Schematic outline of functional therapeutic approaches for stem cell based-therapy.** Development of therapeutic approaches for stem cell-based therapy in consideration of the parameters to be optimized and validated for beneficial clinical outcome. The preliminary approach of the selection of most potential stem cell source (autologous or allogeneic) is followed by characterization for the physicochemical, biological, pharmacokinetics and pharmacodynamics properties of these cells at *in vivo* and *in vitro* levels before clinical trials. To further enhance the therapeutic potential of these cells at clinical trial stage, certain concepts from pharmaceutical drug therapies like preconditioning, co-administration, route of delivery, dose and duration of treatment is to be optimized and validated for more effective outcomes. Therapeutic benefits like bioavailability, bioequivalance, targeted delivery and dose associated disease response are to be anticipated by such clinical approaches to enhance the efficacy of the treatment.

Despite the success of several clinical trials using stem cell-based therapy, there are findings reporting the failure in treating diseases [41–45]. Infact, many questions about this technology associated with the best cell type, suitability of homogeneous or heterogeneous cell mixture, delivery method, or route of administration still remains unclear or are under intense investigations. A detail that is often overlooked in the process of stem cell-based therapy is the discrepancy and inadequate cell articulation in treating many of the ailments [46]. Perhaps, this inconsistency might be due to the inappropriate functionalized strategies deployed for therapeutic effects. This severely compromises the potency of the transplanted cells, thereby, limiting their beneficial effect. Hence, to overcome this constraint, we propose that the efficacy of stem cell-based therapy could be enhanced to the highest level by following some of the basic approaches followed in synthetic drug usage. Thus, we hypothesize that the development of strategies from drug therapies pertaining to co-administration, mode/route, site, doses, duration and also the preconditioning of stem cells will allow the restoration of the functionality in injured or diseased organ/tissue and also to decrease unanticipated clinical effects (Figure 1). This approach could be helpful for fulfilling the potential promise of this most exciting power of body’s own repair kit to cure diseases.

### Strategies to increase the efficacy of stem cell therapy from drug therapies

Considering the most pressing cell therapy challenges, development of innovative pathways is highly recommended because conventional approaches have some limitations as far as efficacy is concerned. To address these challenges, concepts from pharmaceutical drug research is highly recommended to harness the power of stem cells for improved, long-lasting outcomes. The success in pharmaceutical research is highly dependent on the evaluation of the drug-disease relationship. The process of following such relationship is attained by a series of therapeutic interventions, which requires the identification of a broad range of biomedical data sources. Such accumulating evidence has led to the adoption of certain key approaches in cell therapies from lessons learned in pharmaceutical drug setting.

### Co-administration

In clinical practices, doses or levels of therapy need to be tuned based on the severity of the illness or other metabolic characteristics of the patient’s underlying condition. Employing a range of therapies could provide physicians with the flexibility to formulate an effective therapy which is tailor-made for the patient because it takes into consideration the nature and stage of the disease progression. In this regard, there is now an increasing interest in the co-administration of drugs or biomolecules which are often reformulated into fixed-dose combinations [47]. The benefits are numerous; for example, drugs can be used in lower concentration which may diminish both the side effects and the treatment costs. Additionally, treatments can be personalized to individual needs [48]. Ye et al. [49] and Rosanio et al. [50] have previously drawn attention to the benefits yielded from the co-administration of low-dose atorvastatin drug when combined with other agents that targeted the specific pathological conditions associated with cardiovascular disease. These researchers demonstrated that co-administration of low dose atorvastatin with either pioglitazone or sildenafil have significantly reduced the infarct size in an animal model with myocardial ischemia-reperfusion injury. These results demonstrated how two different agents may act synergistically, *via* different pathways to activate the same pro-survival targets.

Similarly, we are theorizing here that by co-administrating with different types of stem cells either concurrently or in the course of events can greatly augment the efficacy of stem cell treatment. Our notion is supported by a previous study where co-administration of endothelial and smooth muscle progenitor cells of umbilical cord blood has boosted the efficiency of vessel development in a nude mouse model of critical hind limb ischemia [51]. Additional research also suggested that the combined transplantation of human endothelial cells and mural cells have synergistically improved the blood flow of nude mice of ischemic hind limbs, remarkably, compared to the single cell type transplantations [52]. Several other studies showed the increasing use of hematopoietic stem cell transplantation with umbilical cord blood to treat malignant and non-malignant hematologic diseases [53–57].

In light of these findings, the use of multiple cell types, or genetically engineered cells, or combinations of progenitor cells with cytokines, growth factors, or even clinically proven drugs can greatly increase the therapeutic potential. Further studies to interrogate the molecular mechanism of these approaches would, therefore, give better insight for the formulation of effective treatment.

### Pre-conditioning

Pharmacological preconditioning is potentially a strong therapeutic tool. Preconditioning has shown to be involved in the protective effect from some membrano-tropic drugs on activation of metabolic processes, thereby improving the resistance of cell structures to various stress factors, in particular ischemia (glycolysis, protein synthesis and phosphorylation of membrane proteins etc.) and hypoxia [58–62]. Therefore, pharmacologic preconditioning by various types of drugs, culture conditions and physical stimulus has opened up a new perspective to protect organs or tissues from transplantation-associated injury, thereby enhancing the success of transplantation therapy [63]. Likewise, stem cell preconditioning and programming by physical, chemical, genetic, biomolecules and pharmacological manipulation of the cells has shown promise and “prime” the cells to the “state of readiness” to withstand the rigors of lethal ischemia *in vitro* as well as post-transplantation [64,65]. Genetic programming, however, is considered to be unsafe due to the introduction of foreign genetic material into cells which could lead to tumor formation. Therefore safer reprogramming and programming methods such as recombinant protein-based or histone modification-based reprogramming, which provide alternative safer methods for pre-conditioning stem cells could be an alternative. For instance, the generation of induced pluripotent stem cells using recombinant proteins [66] and use of valproic acid to induce pluripotency to amniotic fluid stem cells is a transgene free approach [67].

One of the major challenges of stem cell transplantation from bench to bedside is to maintain the cell survival hindering the manipulation of stem cells before transplantation. This is due to the pro-apototic signals presence in the culture environment, which acts on the cells upon their dissociation. Preconditioned stem cells and progenitors generally showed improved characteristics such as better cell survival, increased differentiation potential, enhanced paracrine effect, efficient homing and integration at site of injury or diseased tissue/organ upon transplantation [68]. Continuous effort in understanding the biological behaviour during manipulation and bioprocessing of stem cells for transplantation have led to the identification of few small molecules which could mask the cells from apoptotic signals while harvesting them from monolayer. One such molecule is ROCK inhibitor, which has been proven to enhance the survival of the stem cells that undergo long bioprocessing and transplantation procedures [69,70].

Recent studies on cardiovascular diseases have also shown that preconditioning of stem cells (bone marrow-derived MSCs, adipose-derived stroma cells) could play a vital role in cardio-protection and it has also enhanced the therapeutic efficacy invariably. These studies have successfully used a variety of pre-conditioners including: transforming growth factor-alpha [71]; hypoxic condition [72]; stromal-derived factor 1-alpha [73]; mitochondrial reactive oxygen species [74]; trimetazidine (1-[2,3,4-trimethoxybenzyl] piperazine [75]. Hence, preconditioning could be suggested as a viable option for overcoming one of the critical barriers of stem cell therapy such as the rapid decline in viability and function of the transplanted cells, which otherwise greatly compromise the potency of those transplanted cells [76,77]. Nevertheless, cellular preconditioning could enhance all the trophic mechanisms (intracrine, autocrine, and paracrine signals), the expression of survival signalling molecules, and microRNAs, which could confer the cytoprotective effect [77]. Selective upregulation of survival and protective molecules like hypoxia-inducible factor-1 (HIF-1), trophic/growth factor, Protein Kinase B (PKB/AKT), focal adhesion kinase (FAK), extracellular signal-regulated kinase (ERK), glycogen synthase kinase-3ß (GSK-3ß), matrix metalloproteinase-2 (MMP-2), survivin and Bcl-2 are responsible for protective signalling in response to preconditioning stimuli [78,79]. One of the documented mechanisms of pharmacological preconditioning explains the significant role of mitochondria in cytoprotection by preventing mitochondrial permeability transition pore induction [78]. This is achieved by activating mitochondrial ATP-sensitive potassium (mitoK_ATP_) channels by preconditioning agents, which attenuates the mitochondrial Ca^2+^ overload thus preventing mitochondrial permeability transition pore induction. Additionally, preconditioning can induce the upregulation of heat shock proteins, which facilitates translocation of gap junction proteins like connexins Cx43, an important mediator between adjacent cells in the form of hemichannels. Opening of Cx43 hemichannels helps in early communication between the transplanted and host cells [78,80]. This will be of great significant factors to achieve a better communication between cells which will subsequently ease the engraftment and functionality of the transplanted cells at target site.

Another significant element of endogenous preconditioning mechanisms involves the up-regulation of stromal-derived factor −1 (SDF-1) and CXCR4 factors which play critical roles in mobilization, homing and engraftment of stem cells [78,81,82]. The migration and homing efficacy by SDF-1-CXCR4 chemotaxis is activated mainly by focal adhesion kinase (FAK) through JAK2/STAT3 signalling [78]. Alternatively, P13K/Akt/eNOS signalling also have been reported to trigger the SDF-1 mediated migration of stem cells [78], in which these signalling pathways also play a major role in proliferation and survival of cells. Though found effective, still many of the tissue engineering studies haven’t explored the potentiality of pre-conditioning. Therefore, we emphasise that treating the cells with exogenous agents such as conventional drugs, bioactive molecules, specific growth factor and signalling molecules during *ex vivo* expansion before transplantation could greatly increase the efficacy of stem cell therapy.

### Route of transfusion

Whenever new methods or routes are introduced for synthetic drug administration, it becomes vitally important to gain better insight into their pharmacokinetics and pharmacodynamic implications [83]. For any drug to exert its pharmacological effect, it must first gain its entry into the body, followed by absorption into the blood stream, which finally transports or distributes them to its target of action. However, certain factors are found to influence the process of drug absorption and restrict the efficacy of treatment. These include the physicochemical properties of the drug which determine transfer across cell membranes, formulation or physical state of the drug, site of absorption, circulation at absorption site and area of absorbing surface [83]. Absorption site or the port of entry, the speed, ease, and degree of absorption are determined by the route of drug administration [83].

Similarly, a better understanding on the route of administration for efficient viable cell delivery becomes a necessity for stem cell therapies [84,85]. However, little is known regarding the optimal delivery strategy for stem cells due to the inadequacy of the tools to label and track those transplanted cells. To date, depending on the patient condition, a variety of stem cell delivery options have been adopted based on preclinical and clinical studies for repairing different tissues like cardiac tissue (intracoronarily, intramyocardial, catheter-based injections) [86–90]; brain (intracranial, intravenous, intranasal, circulatory systems) [91–93] and spinal cord (intra-arterial, intravenous; cerebrospinal fluid by lumbar puncture) [94,95]. However, several studies have revealed that route of stem cell (MSCs) administration near to the target site have shown more potential than the other routes of administration [96–99]. In some other studies multiple route delivery technique has shown substantial advantage over the single route administrating techniques, to ensure the cell homing to the target area to promote tissue repair [100]. Although, the complexity of multiple route delivery limits the mechanistic understanding of cell- based applications, the feasibility of safe and assured delivery to the target niche ensures the endogenous repair which is greatly demanding criterion, due to heterogeneity of human injuries. Hence, for the success of any stem cell therapy, we suggest that finding the right route for cell administration is of prime importance. This is only achievable by understanding the migratory property of these cells and the cell behaviour along the migratory route. Moreover, it has to be made sure that the transplanted cells are not homing, other than the area of interest of organ repair, otherwise it could lead to disturbance of normal homeostasis in the function and regulation of other organs [57]. Therefore, we propose that the approach of stem cell therapy should be basically target specific and the cell administration to achieve this target specificity [101]. Thus, developing tools to label and track those transplanted cells, prime the cells to adopt the migration potential and co-administrate factors to effectively induce the repopulation of integrated stem cells would be of great benefit to make cell therapy a success.

### Treatment modalities: doses and duration

Every patient has a unique therapeutic threshold for each prescribed drug due to individual drug sensitivities [102,103]. Age and gender, are two other significant factors which influence the pharmacokinetics and pharmacodynamics of a drug [104,105]. Hughes and Aronson [106] defined that duration of action of a drug is directly proportional to the logarithm of dose for a wide range of different drugs, revealing the significant role played by both these variables on the treatment outcomes. Therefore, when drugs are administrated, a meticulous understanding of the dose–response relationship is of great significance for achieving the specific therapeutic effect while minimizing their side-effects [107,108]. Generally for stem cell based treatment, a 70 kg-patient needs approximately 2 × 10^6^ MSCs per kilogram body weight for transplantation [109]. However, reports suggest that there are variations in optimal or effective doses required for various diseases, albeit the source of the stem cells is the same. For examples, 3–5 × 10^7^ cell/kg ex-vivo expanded autologous bone marrow derived mesenchymal stem cells (BM-MSCs) were administrated into each patient with multiple sclerosis [110] whereas in spinal cord injury, 5–6 × 10^6^ cells/kg were transplanted [111]. Liang et al. [112] reported that a dose of 1 × 10^6^ cells/kg allogeneic bone marrow MSCs were transplanted *via* intravenous infusion through a single administration for treating refractory systemic lupus erythematosus. Clinical trials for graft-versus-host disease (GVHD) using allogeneic MSCs (adipose derived stem cells, bone-marrow derived MSCs, hematopoietic stem cell, peripheral blood stem cell, Prochymal) demonstrated beneficial responses in both pediatric and adult patients when infused (single to multiple times) with different doses based on severity of the disease [1]. Similar studies of dose escalation phase I clinical trial using different doses of allogeneic MSCs (Prochymal) ranging from 0.5, 1.6, and 5 million cells/kg in patients with myocardial infarction showed safety efficacy data [113]. An overall assessment of the patient health was evaluated during the course of trial for signs of improvement or deterioration. Significantly, when analyzed for dose-dependent effects, patients exhibited dose responsiveness in terms of premature ventricular contraction (PVC). The PVC count did not differ in the control and low dose treatment groups, but variation was evident in the mid and high dose treatment groups. In yet another pilot study, phase I/II clinical trials was conducted for demonstrating the efficacy of allogeneic bone marrow derived MSCs in patients with chronic ishemic cardiomyopathy (ICM) [114]. Currently, phase I clinical trial is being conducted on dose escalation study of autologous MSCs in patients with Amyotrophic Lateral Sclerosis for determining the dose limiting toxicities [115,116]. In another study, dose escalation Phase 2 clinical trial of MultiStem (allogenic cell therapy treatment comprising of multipotent adherent bone marrow cells) in patients with ischemic stroke was measured with two dose tiers of 400 million and 1200 million cells per patient to determine the highest well-tolerated and safe single dose of multistem [117]. Further clinical trials are being conducted for dose-escalating therapeutic study of allogeneic bone marrow derived mesenchymal stem cells for the treatment of fistulas in patients with refractory perianal crohn’s disease [118].

While study results provide assurance for the safety of allogeneic human mesenchymal stem cells (hMSCs), further work is in demand to delineate the mechanism of action of this MSC therapy in dose responsiveness manner. In addition, other factors such as source of stem cells, their functionality level, disease stage and route of administration, niche microenvironment can also influence the dosage and duration of stem cell therapy for a particular disease. For example, studies have reported poor engraftment of transplanted stem cells which could be due to impaired target niche microenvironment after intensive chemoradiotherapy or lower doses of cell infusion [57]. Hence, development of dose-intensive delivery programs can possibly improve the response rates and outcome of treatment. Further evaluation of the efficacy in terms of safety and functional outcome of repeated dose/duration period can result in major advance in stem cell treatment strategies when compared to conventional therapy.

**Figure 2:**
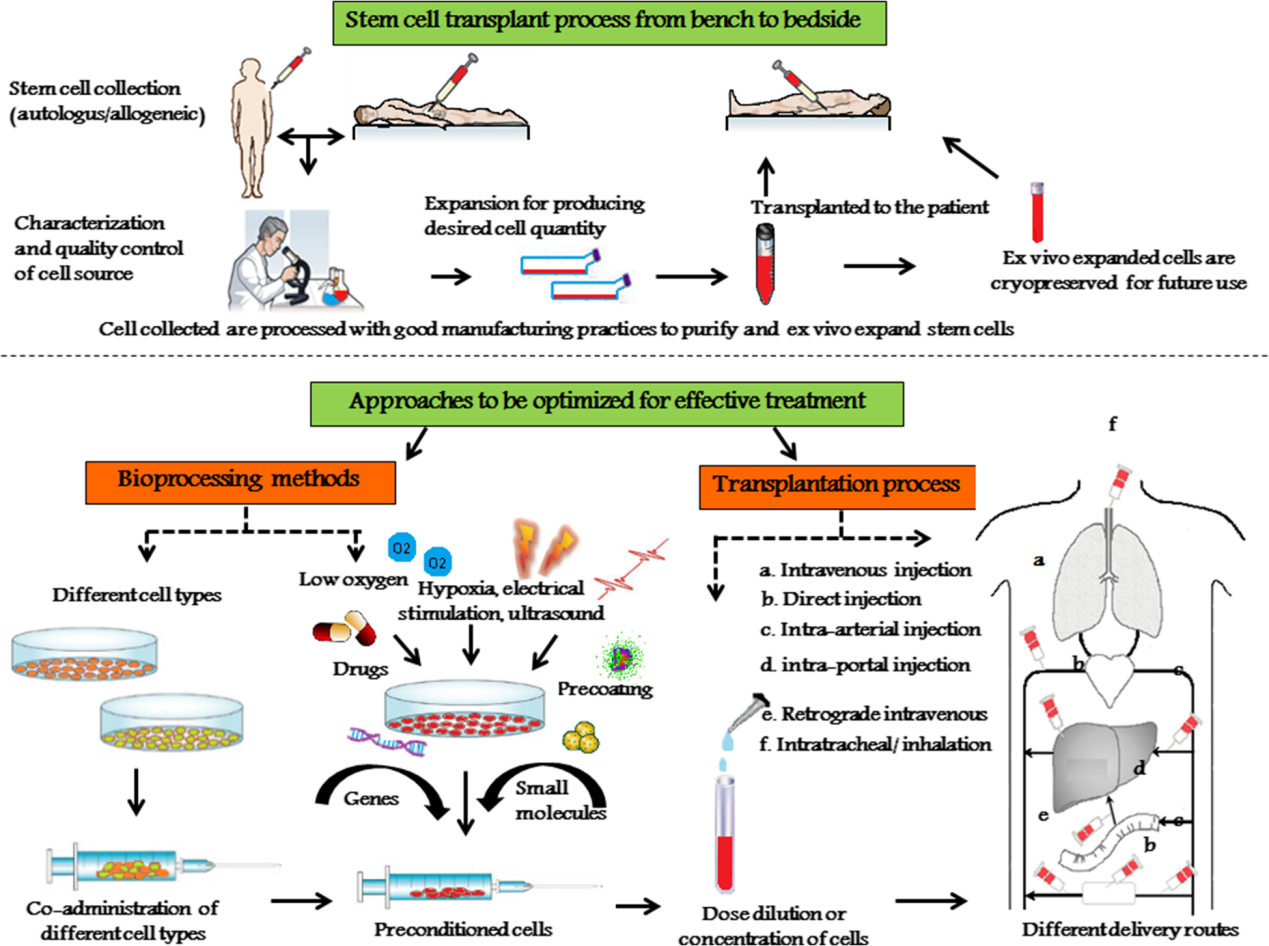
**Model stem cell transplantation process for effective personalized application.** An overview of stem cell transplantation model tailored-made for a personalized therapy, with ideal culture conditions to be optimized at bio-processing and transplantation procedures to enhance the viability, migration and homing properties of the transplanted cells for achieving the best possible effective personalized therapy.

Although, promising results have been highlighted using MSCs in clinical trials, the underlying dose and follow up dose associated mechanisms still remains unclear. Nevertheless, studies involving MSC therapy demonstrates a clear involvement of intricate mechanism of migration, homing efficiency, immunological properties, differentiation and secretions of bioactive molecules in an integrative manner. Taking all these notes together, it is possible that dose associated potential efficacy of allogeneic/autologous stem cells with single or repeated infusions can translate significant changes in clinical aspects. Further, it is plausible to propose that treating a disease with terminally differentiated cells would require higher cell number than by progenitors because the latter option provides the opportunity for this cell to proliferate and produce a larger mass and may differ in its secretions of bioactive molecules to substitute or repair the lost functional tissue. However, the unique tropism of cell therapy has to be taken into account for the optimization of cell infusion doses and duration to prevent cell losses for an effective transplantation setting. Taking all these issues together, stem cell therapy has a great potential to evolve further answers to the challenges associated with cell therapy transplantations as to ensure reproducible personalized therapy (Figure 2).

### Therapeutic road map differ between autologous or allogeneic (off the shelf) cell products

Globally there are between 300 to 700 companies, ranging from small university spinouts to multinational corporations involved in developing cell-based therapy such as GlaxoSmithKline, Johnson & Johnson, Pfizer, and Sanofi (Genzyme) [119]. Persistent development of cell-based therapy research and development (R&D) industrial players in stock market index suggests the demand and potential of this industry as a distinct healthcare sector (119) Table 1. Clinical transplantation mostly set to utilize allogeneic or syngeneic (genetically similar) donor cells due to the complications/difficulties in extracting or obtaining ample patient-derived cells. One main reason for this phenomenon is that aging and disease pathophysiology affect the number and functional properties of stem cells in patients [3,120]. Besides, allogeneic cells can be readily isolated from healthy donors and thus can be used as an “off-the-shelf” biological reagent. Furthermore, pre-cultured human stem cells offer an improved practicality in consideration for cell therapy than compared with on-site isolated autologous cells. Firstly, the homing ability of allogeneic MSCs to injured site or tissue could be well stimulated during culture by manipulating the expression of SDF-1/CXCL12 axis, which in turn induces the migratory ability [113]. Second, the lack of various major histocompatibility complexes (MHC) and co-stimulatory cell-surface antigens makes them a perfect allogeneic graft, additionally having the conferred anti-inflammatory properties [113]. For instance, the host immune system cannot detect the donor MSCs (allogenic graft) on account of low levels of human leukocyte antigens (HLA) present on surface of these cells [1]. Third, it offers an enriched population of therapeutically relevant cells, as demonstrated in various pre-clinical studies [113]. Finally, the differentiation potential of stem cells into tissue specific cells of different lineages is advantageous [1].

**Table 1:** **Stem cell therapy companies and their cell products in development**

**Company name**	**Cell types**	**Therapeutic programs/product name**	**Clinical area**	**Reference**
Aastrom Bioscience	Autologous	Multicellular therapy **(Ixmyelocel-T)**	Cardiovascular disease	[121]
			Peripheral artery Diseases (PAD)	
Cytomedix	Autologous adult stem cells	**ALDHbr** (autologous regenerative cell therapy utilizing proprietary ALDH Bright Cell technology to isolate biologically active pluri-potent stem cells for therapeutic use)	Ischemic heart failure	[122]
			Ischemic Stroke	
			Critical limb ischemia	
			Peripheral artery Diseases (PAD)	
Cytori Therapeutics	Autologous adult adipose derived stem cells	**Celution 800/CRS system** (The system automates and standardizes the extraction and concentration of patients own Adipose Derived Regenerative Cells (ADRCs) in a clinical setting, enabling real-time access to autologous medical grade cells ADRCs	Acute MI	[123]
			Cardiac failure	
			Burn care	
	Regenerative cells produced utilizing proprietary		Soft tissue injury	
			Orthopedics	
			Breast reconstruction	
			Sports medicine	
Dendreon	Autologous cellular immunotherapy	**Provenge (sipuleucel-T)** Body’s own immune cells are isolated and reprogrammed to attack advanced prostate cancer.	Prostate cancer (asymptomatic/minimally symptomatic metastatic castrate)	[124]
Fibrocell Science	Autologous Fibroblasts	**Azficel-T s BLA Program**	Skin	[125]
		**Intrexon Synthetic Biology Rare Disease Program** (Autologous cellular product treat rare and serious skin and connective tissue diseases and conditions)	Connective tissue diseases	
Immuno cellular therapeutics	Autologous dendritic cell	**ICT- 107** (newly diagnosed Glioblastoma)	Glioblastoma multiforme	[126]
		**ICT- 121** (Recurrent Glioblastoma)	Ovarian cancer	
		**ICT- 140** (Ovarian cancer)	Cancer stem cells	
		Autologous dendritic cell based vaccines		
Japan Tissue Engineering Company (J-TEC)	Autologous cell	**Autologous cultured epidermis**	Burns	[127]
		**Autologous cultured Cartilage**	Cartilage defects	
		**Autologous cultured Corneal Epithelium** (Tissue engineering using autologous cells)	Corneal damage	
Northwest Biotherapeutics	Autologous dendritic cells	**DCVax technology** (Autologous dendritic cell-based therapy)	Glioblastoma multiforme (brain tumor)	[128]
			Prostate cancer	
Pharmicell	Autologous bone marrow derived MSCs	**Hearticellgram** (Autologous bone marrow derived MSCs)	Acute Myocardial Infarction	[129]
Advanced Cell Technology	Allogeneic adult stem cells hESC-derived cells	**Retinal Pigment Epithelial Cell Program for:** Stargardt’s macular dystropy (SMD)	Retinal Degenerative Conditions	[130]
		Dry Age-Related Macular Degeneration (AMD)	Blood and cardiovascular diseases	
		**Hemangioblast (HG) Program for:** Diseases and disorders of circulatory and vascular system		
Athersys	Allogeneic adult stem cells	**Multistem**	Inflammatory & Immune	[131]
			Neurological	
			Cardiovascular disease	
			Ulcerative colitis	
			Ischemic stroke	
BioTime	Clinical-grade human embryonic stem (hES) cell lines	**OpRegen** (hESC-derived RPE cells for the treatment of macular degeneration)	Age-related degenerative disease	[132]
		**OPC1** (hESC-derived oligodendrocyte progenitors for spinal cord injury);	Spinal cord injury	
		**VAC1** (a dendritic cell-based vaccine for cancer based on the telomerase antigen)	Neuroscience orthopedics blood and vascular diseases oncology	
Medipost	Allogeneic human umbilical cord blood and Umbilical Cord Blood derived Mesenchymal Stem Cells (hUCB-MSCs)	**Cartistem** (cartilage defect)	Cartilage defects	[133]
		**Neurostem** (neuro-degenerative disorders)	Alzheimer’s disease	
		**Pneumostem** (pulmonary disorders)	Amyotrophic Lateral Sclerosis (ALS)	
		**Promostem** (early engraftment of donor hematopoietic stem cells (HSCs))	Stroke	
			Chronic lung disease (premature babies)	
Mesoblast	Allogeneic adult derived mesenchymal precursor cells (MPCs)	**Proprietary Mesenchymal stem cells lineage technology**	Systemic diseases with underlying inflammatory and immunologic etiology	[134]
	Mesenchymal stem cells (MSCs)		Cardiac and vascular diseases	
	Dental pulp stem cells (DPSCs)		Orthopedic diseases of spine	
	Hematopoietic stem cells (HSCs)		Improving outcome of bone marrow transplantation	
Neostem	Autologous adult stem cells,	**Targeted Immuno Therapy Program**	Cancer treatment	[135]
	Allogeneic T cell Embryonic like stem cells & Progenitor Cells	**CD34 cell program**	Ischemic repair	
		**T Regulatory Cell Program**	Immune modulation	
		**VSEL (Very small embryonic like stem cells ) technology**	Tissue regeneration	
NeuralStem	Human hippocampus Neural Stem cells	NSI-189 (a compound developed for oral administration for psychiatric and cognitive disorders)	Amyotrophic Lateral Sclerosis (ALS)	[136]
			Traumatic brain injury	
			Alzheimer’s disease	
Osiris Therapeutics	Autologous and Allogeneic Mesenchymal stem cells (MSCs)	**OvationOS** (bone matrix designed for the filling of bony voids and to support bone repair and regeneration)	Bone damage	[137]
			Soft tissues(cartilage and tendon) wound healing	
		**Grafix (**Allogeneic MSCs in extracellular matrix)		
		**Cartiform** (3-dimensional architecture of hyaline cartilage that contains the necessary cellular and molecular components for articular cartilage repair, and is primed for mesenchymal stem cell (MSC) activity)		
Pluristem therapeutics	Allogeneic cells	**PLX** (Placental expanded cells)	Cardiovascular disease	[138]
			Orthopedic disease	
			Pulmonary diseases	
Stemcells	Allogeneic tissue derived adult stem cells and progenitor cells	**HuCNS-SC** (human neural stem cells)	Spinal cord injury	[139]
		**hLEC** (human liver engrafting cells)	Peripheral artery Diseases (PAD)	
			Pelizaeus-Merzbacher Disease (PMD)	
			Age-related macular degeneration (AMD)	
Tigenix	Autologous and Allogeneic adult stem cells	**ChondroCelect** (Autologous Chondrocytes)	Cartilage defects	[140]
		**eASCs** (Allogeneic expanded stem cells extracted from adipose tissue )	Perianal fistulas	
			Rheumatois arthritis	

Although the differentiation potential of embryonic stem (ES) cells is greater than of somatic stem cells, studies have proven that MSCs can be conferred into more pluripotent state by epigenetic modification which facilitates an efficient differentiation into cells of different lineages [1]. Hence, this approach makes MSCs suitable for the treatment of various disease pathologies and injuries.

**Figure 3:**
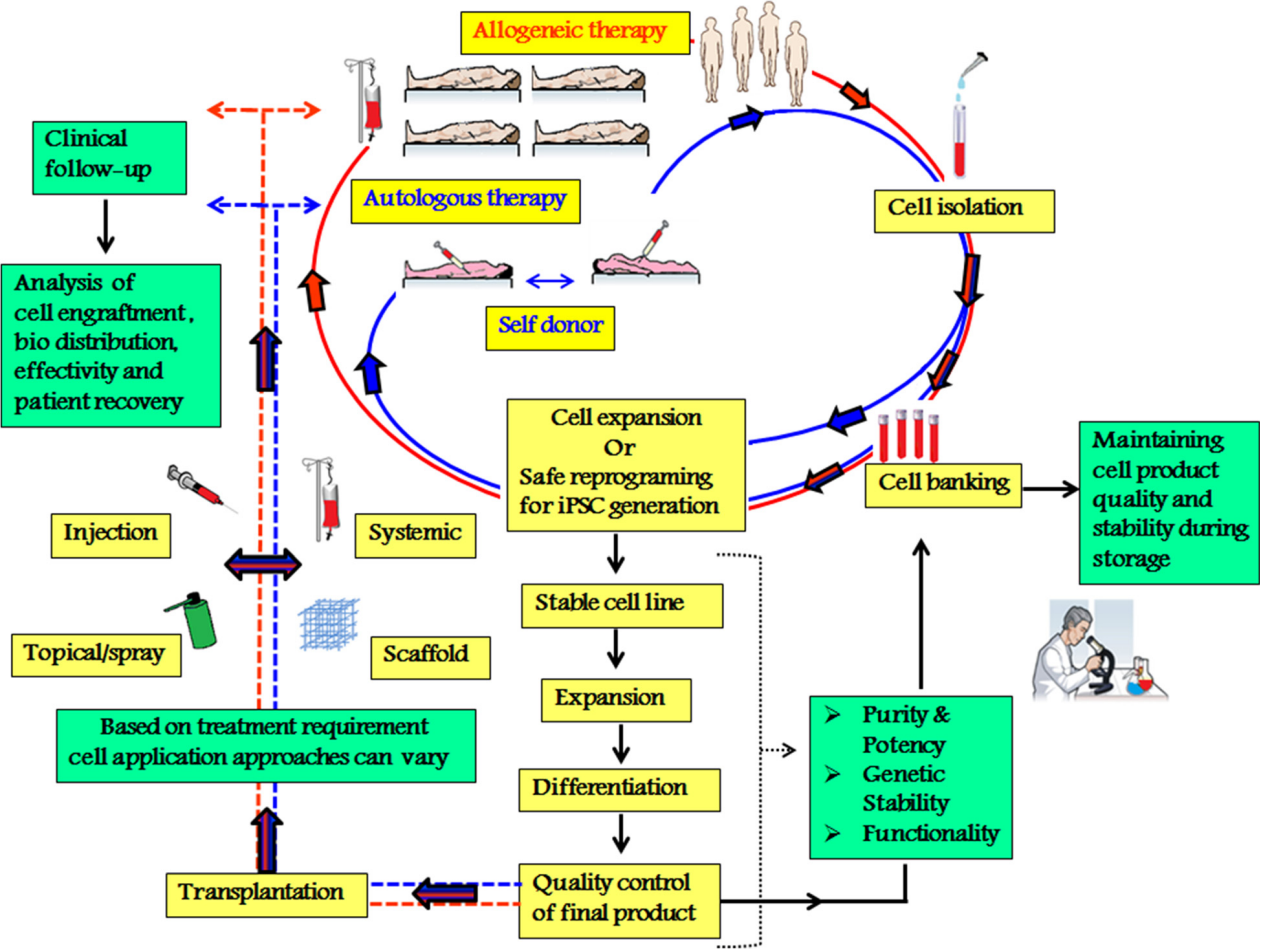
**Road map for allogeneic and autologous “off-the-shelf” cell products for therapeutic applications.** Road map for allogeneic and autologous “off-the-shelf” cell products for stem cell transplantation. Isolated cells can be used for the cell therapy with or without the manipulation of the cells for enhancing the potentiality or differentiate them into desired cell types before the transplantation. These cells could be stored for the future personalized medical applications. The cell transplantation approaches can vary from systemic and injection to topical application or with scaffold depending on the treatment requirement of the patient. Finally the stem cell transplanted patients should be followed-up of analyzing the disease recovery and treatment effectivity.

In some instances, there are studies addressing the safety concerns associated with the preparation of allogeneic cells, which showed a detectable level of HLA once they are integrated and differentiated in the host. This leads to the graft rejection and chronic immune responses [141]. Therefore, issue over such graft rejection even when the cells expresses HLA at low level, in turn makes the autologous and minimally *in vitro* manipulated cells as an attractive and promising curative bio-reagents for many regenerative, anti-inflammatory and autoimmune related diseases. Indeed, studies have revealed the suitability of both autologous and allogeneic culture-expanded MSCs in cardiovascular studies [114], in which direct myocardial injection of autologous expanded BM-MSCs was found to show significant effect in structural and functional measures for ischemic Left Ventricular dysfunction [142]. While the attempt to utilize the autologous MSC to achieve individual treatment has been the focus for the past few decades, application of these cells through fibrin spray or in the form of conditioned media (culture media of MSCs consisting of cell secretions) are also under investigation for treatments like wound repair, burns and other soft and connective tissue repair [143–145]. Having said this, factors such as nature of the disease, type of stem cells and patient condition need to be taken into consideration before formulating an effective procedures and approach to utilise autologous and allogeneic cell-based therapy (Figure 3).

A great new venture in which the cell therapy based industry moving towards is the generation of patient specific induced-pluripotent stem cells (iPSCs), bringing a whole new dimension in developing cell based therapy to the next level moving this endeavour from bench to bedside application. Various aspect of biological properties and therapeutic models are under investigation in order to develop such patient specific stem cells. Although, a recent study has reported limited immunogenicity of transplanted cells differentiated from iPSCs and embryonic stem cells [146], the immunological barriers of patient specific- cell transplantation are the same as those encountered and continue to confound for allogeneic cells. Indeed, long term culture, genomic instability, interference with matrix structure, genetic manipulation and epigenetic reprogramming can impair immune privilege status of the autologous and allogeneic cells [147]. Accordingly, in such safe personalized regenerative stem cell therapy scenarios, the selection of the least incompatible stem cells, safe generation of stable and clinical grade iPSCs lines in xeno-free and fully defined culture conditions and proper characterization of quality parameters to ensure cell purity and potency along with the host MHC information is to be considered to avoid or attenuate the host immune response to the transplanted stem cells [147,148].

Another approach would be the development of HLA-matched cell banks, which are the focus of stem cell biobank services provided by some of the healthcare industrial players [119]. Considering the surrounding merits of using autologous and allogeneic stem cells, it might be further insightful to gain beneficial information on the immunogenetics by direct comparison between both these cell types and iPSCs and iPSC-derived therapeutically relevant cells to control and ensure successful therapeutic impact upon transplantations. Thus, the use of “off the shelf cell products” is highly attractive in their point-of-care in cell therapeutics. Hence, by ensuring the continued development of the regulatory framework, therapeutics interventions and other supportive technologies by healthcare bodies and industries, it will accelerate this exciting new therapeutic venture and bring impact to the future successes of the cell therapy industry.

### Pharmaceutical drug therapeutic lessons translatable and not translatable to stem cell therapies

Both synthetic and natural drugs are generally well-characterized of their formulation, biochemical reactions, pharmacokinetics, pharmacodynamics and predictable end and by-products. On the other hand, the significant limitations to the development of stem cell therapy for patients includes; a) the lack of knowledge on the active cellular constituent of stem cells that are responsible for the reparative action b) the possibility that therapeutically active cells may represent only a very small fraction of the total stem cell population and, c) their potential to differentiate into variety of cell lineages govern by the *in vivo* microenvironment factors. Mostly, pharmaceutical treatments deliver a single agent at a specific dose to either catalyse or inhibit the biochemical reactions. On the other hand, stem cells are site-regulated and they are capable to secrete bioactive factors and give signals at variable concentrations in response to local microenvironmental cues [141]. Thus, while designing cell therapy, the criteria, measurement and outcome one would expect for an optimal combination to specific organs or disease could most likely be variable because of the complexity of the parameters. Hence, understanding the complexity represented by the behaviour and responses of these stem cells requires the development of novel approaches and strategies which are not present in drug therapy and yet to be developed.

Albeit therapies based on drugs and stem cells have to be paved in a different way. Certain critical curative concepts of drugs therapies like the pharmacogenomics will be of high importance in developing cell therapies. In pharmacogenomics, it has been hypothesized that human genetic variations could dictate the efficacy and toxicity of drugs [149]. Thus the genetic information can be utilized to predict the safety, toxicity and/or efficacy of drugs. Accordingly, pharmacogenomics studies are mostly carried out through candidate gene approach and prior knowledge of the drug mechanism. Specific genes that encode for drug metabolizing enzymes, drug transporters and drug related proteins are also considered for screening before treatment. Similarly, cell secretions have a role in an individual’s genotype expression [120]. Equivalently, to commensurate the cell therapeutic mechanism for such response reaction could be analysed for the genes of interest and also other relevant genes involved. For instance, those genes involved in the immune response could be used. Thus, the concept of utilizing the role of human genetic variation to such responsive agents (either stem cells or drugs), provide credence to the concept of personalized medicine.

To better understand these factors and to optimize the beneficial effect of these therapies, it is important to monitor the clinical trials and the outcomes. While the best treatment strategy either by employing conventional drugs or stem cell transplant adds to the socioeconomic considerations, in other situations research investigating the psychosocial experience and quality of life of patients undergoing such treatments should also be considered. Clearly, the beneficial effect of these therapies must be weighed against the risks, but experimental studies must be encouraged to design combinational procedures by exploring newly developed concepts and protocols.

### Future perspective of stem cell therapy in regenerative medicine

Regenerative medicine and cell therapy are poised to have a tremendous impact on the future of medicine by delivering more effective, long-lasting, safe and cost-effective therapies for life- threatening and life-altering conditions than are currently available today. The extent to which we are able to achieve effective cell therapies will depend on assimilating a rapidly developing base of scientific knowledge with the practical considerations of design, delivery, and host response. Hence, a continuous effort is required to achieve a refined application of stem cell therapy to expand the role of stem cells for their vanguard uses. Table 2 details the cell therapy strategies, benefits and current challenges to be refined for more effective stem cell therapeutic procedure.

**Table 2:** **Strategies to be refined to expand the stem cell therapeutic procedure**

**Functional therapeutic approaches**	**Therapeutic strategies**	**Benefits/outcomes**	**Problems to be addressed**
**Co-administration**	Multiple cell types	Utilization of cell heterogeneity	Utilization of correct combinations
	Genetically engineered cells		
	Combinations of progenitors		
	Cytokines	Exploiting cell signalling synergistically	Understanding cell signalling for effective cell formulations
	Growth factors		
	Transcription factors		
**Pre-conditioning**	Chemical (drugs)	Cyto-protection	Developing safer reprogramming & programming methods
	Physical Stimuli (hypoxia, electrical stimulation, ultrasound)	Better cell survival	
	Genetic	Increased differentiation potential	
	Small molecules	Enhanced paracrine effects	Understanding the mechanisms triggering the cytoprotective and other signalling pathways in response to preconditioning stimuli
	Pre-coating	Efficient homing & integration	
**Route of transfusion/translation**	Local or systemic	Targeted delivery	Developing efficient labelling for cell tracking post transplantation
	Intracranial, intranasal, circulatory system	Better cell survival & integration post transplantation	Complexity of multiple route delivery mechanisms
	Intra-arterial, intra venous, cerebrospinal fluid by lumbar puncture		Understanding the migratory properties & migratory behaviour of cells through different delivery routes
**Doses & Durations**	Cell dose based on MSCs sources	Minimize side effects by fixed dose	Determine the potential cell source for transplantation
	Single or multiple infusion	Preventing cell lose	Determine the dose associated efficacy of allogeneic/autogenic/cryopreserved MSCs

## Conclusion

This hypothesis is aimed to propose the possible mimicking or recapitulation of certain clinical approaches from conventional drug therapy to stem cell-based therapy to improve their efficacy. Adopting such approaches will give immense value in translating stem cell therapy from bench to bedside. In addition to satisfying the scientific basis of stem cell treatments, this approach will bring about viable options to overcome current limitations in stem cell therapy. Implication of this approach in stem cell therapy is expected to not only modulate the outcome of intended therapy, but also will be a feasible option for the cost effectiveness of stem cell-based regenerative therapies in the near future. Therefore we suggest that the stem cells therapy should be also be seen from the point of view drug.

## Abbreviations

ESCs: Embryonic stem cells
MSCs: Mesenchymal stem cells
MPCs: Multipotent stem cells
PC: Projenitor cells
SSC: Somatic stem cells
HIF-1: Hypoxia-inducible factor −1
Akt: Protein kinase B (PKB)
CXCR4: C-X-C chemokine receptor type 4 also known as fusin or CD184
FAK: Focal adhesion kinase
ERK: Extracellular signal-regulated kinase
GSK-3ß: Glycogen synthase kinase-3ß
MMP-2: Matrix metalloproteinase-2
mitoK_ATP_: mitochondrial ATP-sensitive potassium
SDF-1: Stromal-derived factor −1
GVHD: Graft-versus-host disease
iPSC: Induced pluripotent stem cells
ICM: Ishemic cardiomyopathy
MHC: Major histocompatibility complexes
HLA: Human leukocyte antigens
SDF-1: Stromal derived factor −1
PAD: Peripheral artery diseases
ADRCs: Adipose derived regenerative cells
ALS: Amyotrophic lateral sclerosis
AMD: Age-related macular degeneration
PMD: Pelizaeus-merzbacher disease
PLX: Placental expanded cells
hLEC: Human liver engrafting cells
HuCNS-SC: human neural stem cells
